# Supplementary material for: Enzymatic Synthesis of a Novel Coumarin Aminophosphonates: Antibacterial Effects and Oxidative Stress Modulation on Selected E. coli Strains
Source: Int J Mol Sci. 2023 Apr 20;24(8):7609. doi: 10.3390/ijms24087609 (PMC10146307; doi:10.3390/ijms24087609)
Supplement: Supplementary file 1 [file ijms-24-07609-s001.zip › ijms-2299476-supplementary.pdf]

Supplementary materials

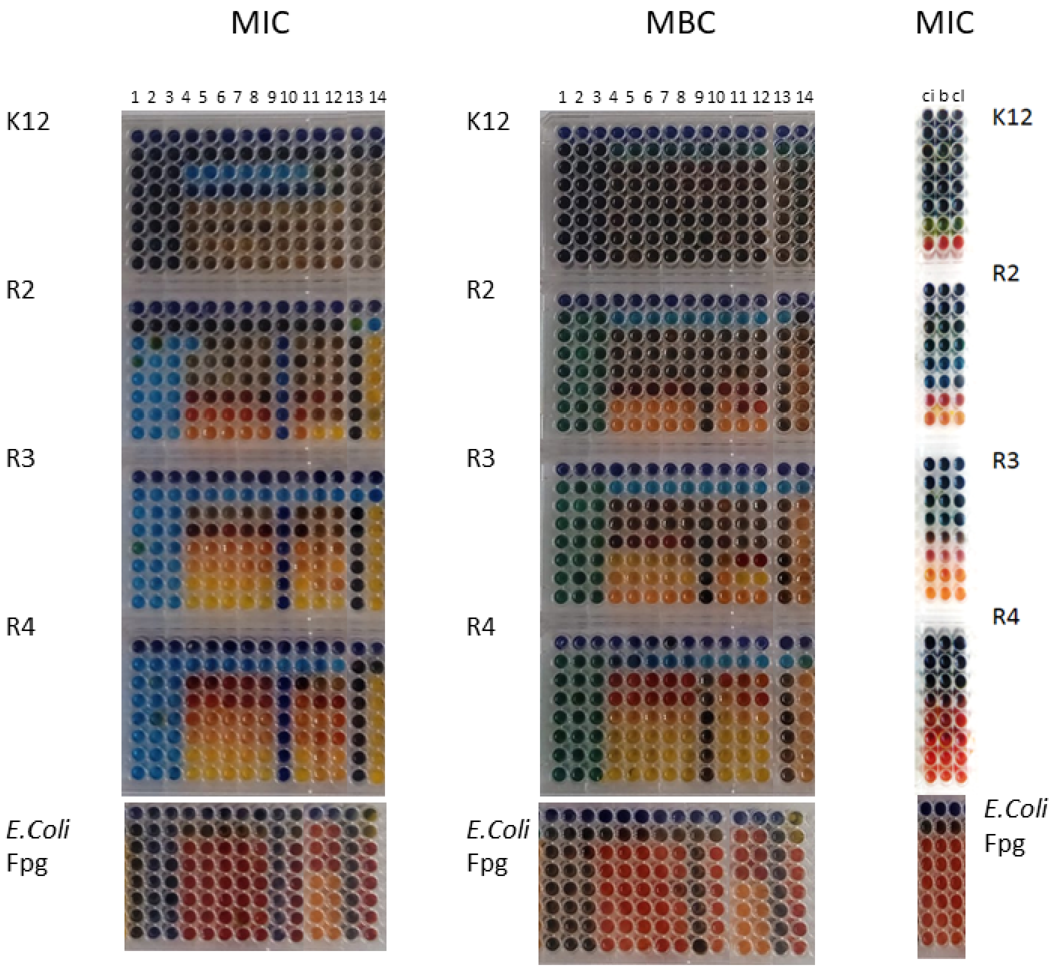

Figure S1. Examples of MIC and MBC on microplates with different concentration of studied compounds (μg/mL⁻¹). Resazurin was added as an indicator of microbial growth with K12, R2, R3, R4 strains and *E.coli* Fpg with tested compounds, as described in Figure 4 and 5 . Additionally, examples of MIC with different strains K12, R2, R3, R4 and *E.coli* Fpg of studied antibiotics with ciprofloxacin (ci), bleomycin (b), and cloxacillin (cl) in (μg/mL⁻¹).

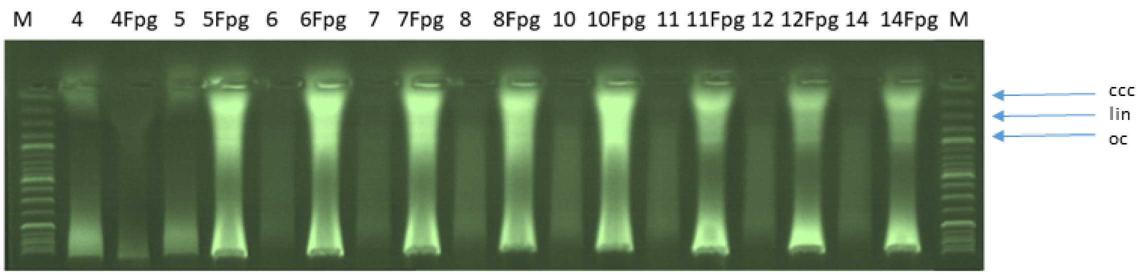

Figure S2. An example of an agarose gel electrophoresis separation of isolated plasmids DNA on R4 strains modified with selected coumarin derivatives and digested with repair Fpg protein. M = marker.

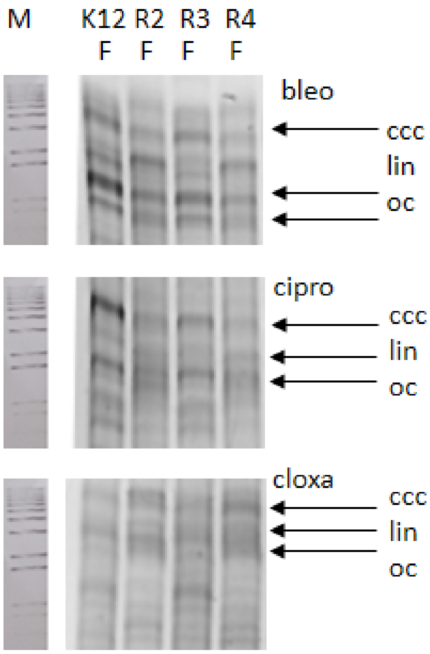

Figure S3. Example of an agarose gel electrophoresis separation of isolated plasmids DNA from K12 and R4 strains modified with antibiotics: bleomycin, ciprofloxacin, and cloxacillin digested (or not) with repair enzymes Fpg. M = marker.

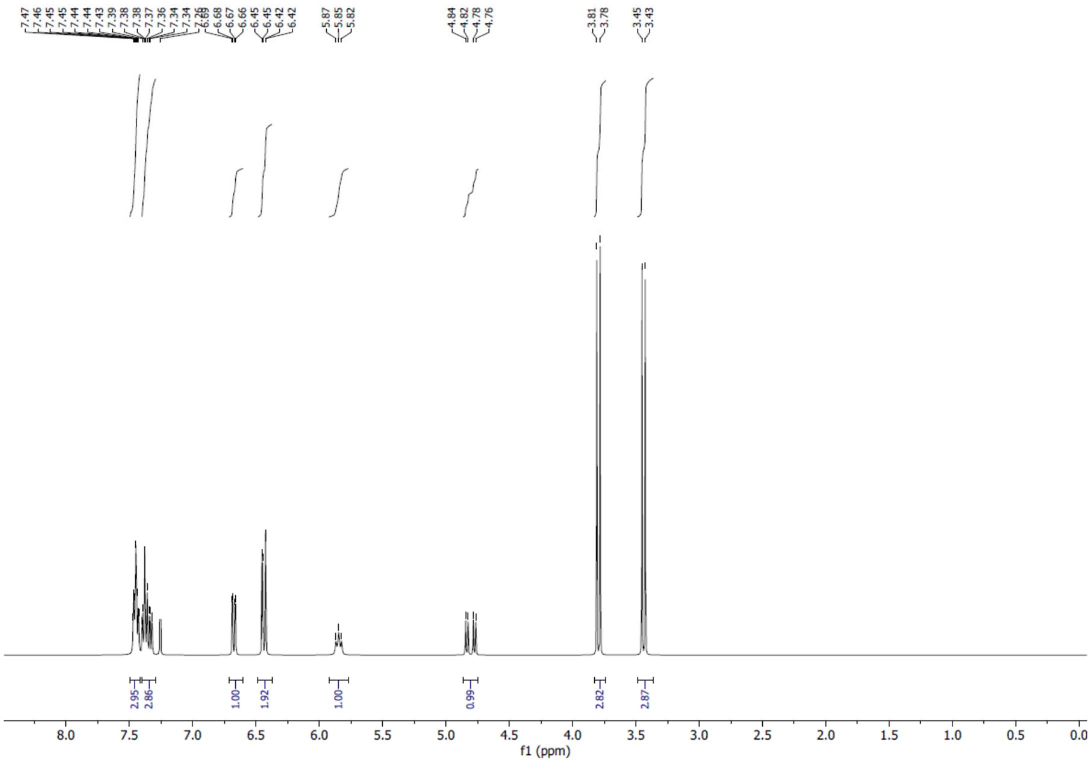

Figure S4. ^{1}H NMR (400 MHz, $CDCl_3$) of (**1**).

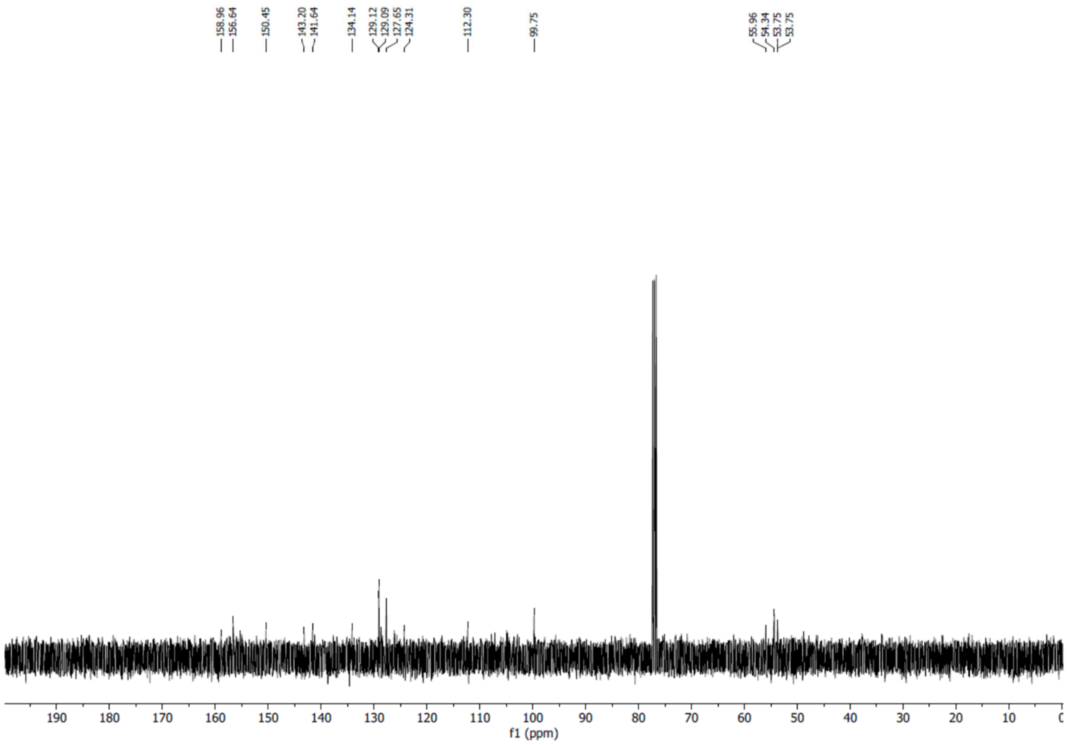

Figure S5. ^{13}C NMR (100 MHz, $CDCl_3$) of (**1**).

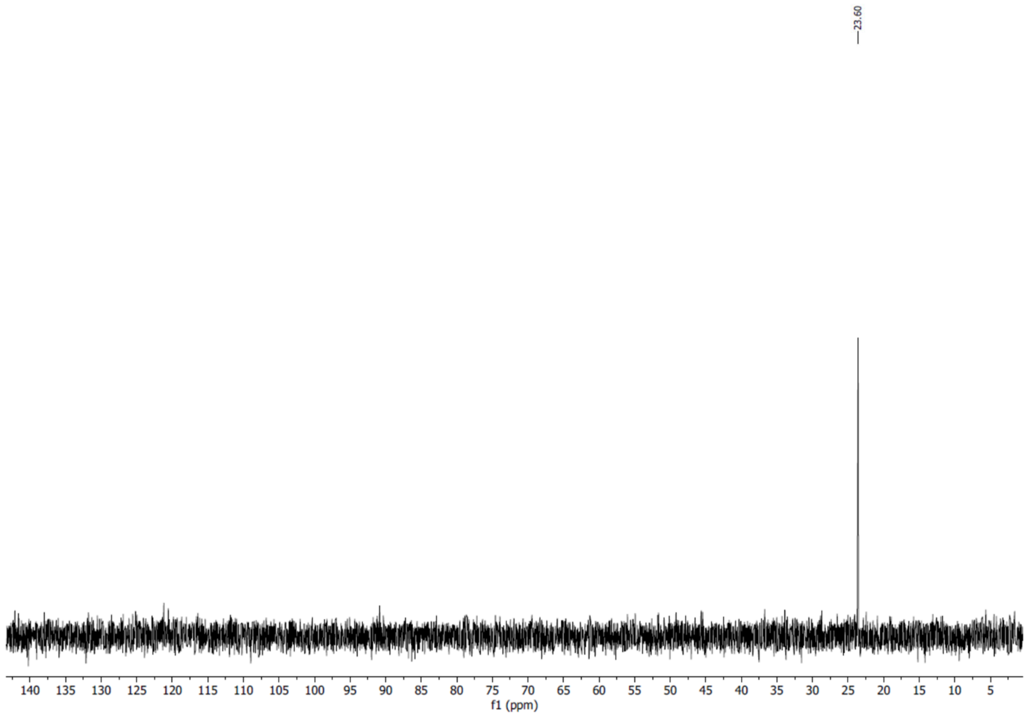

Figure S6. ^{31}P NMR (162 MHz, $CDCl_3$) of (**1**).

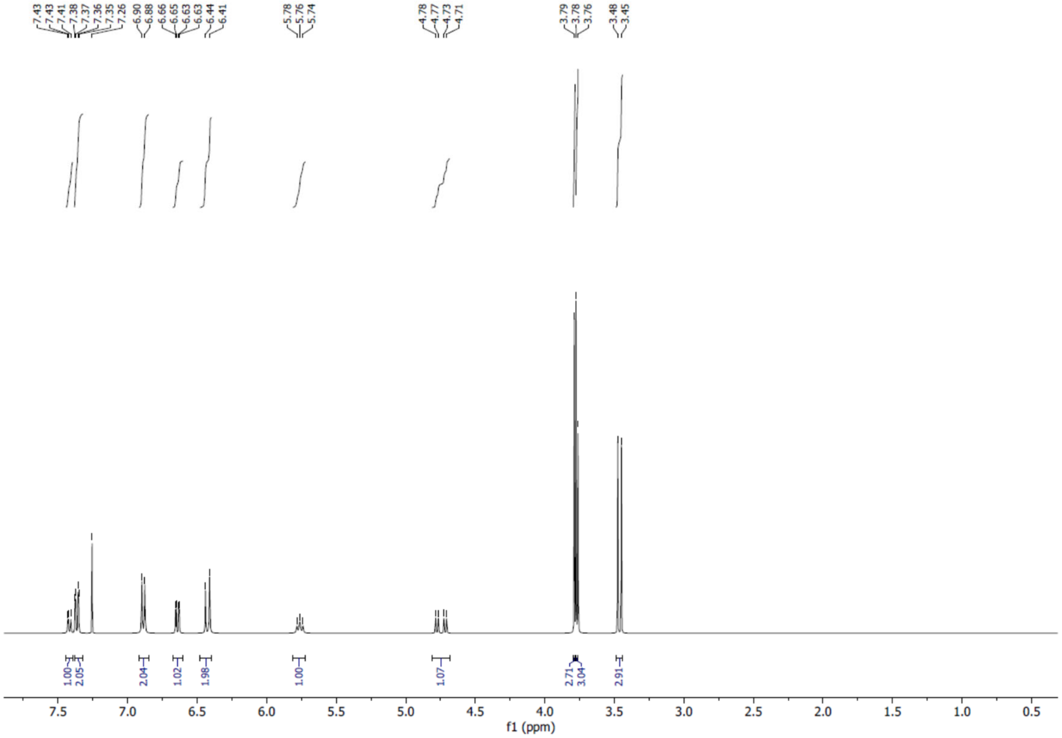

Figure S7. ^{1}H NMR (400 MHz, $CDCl_3$) of (**2**).

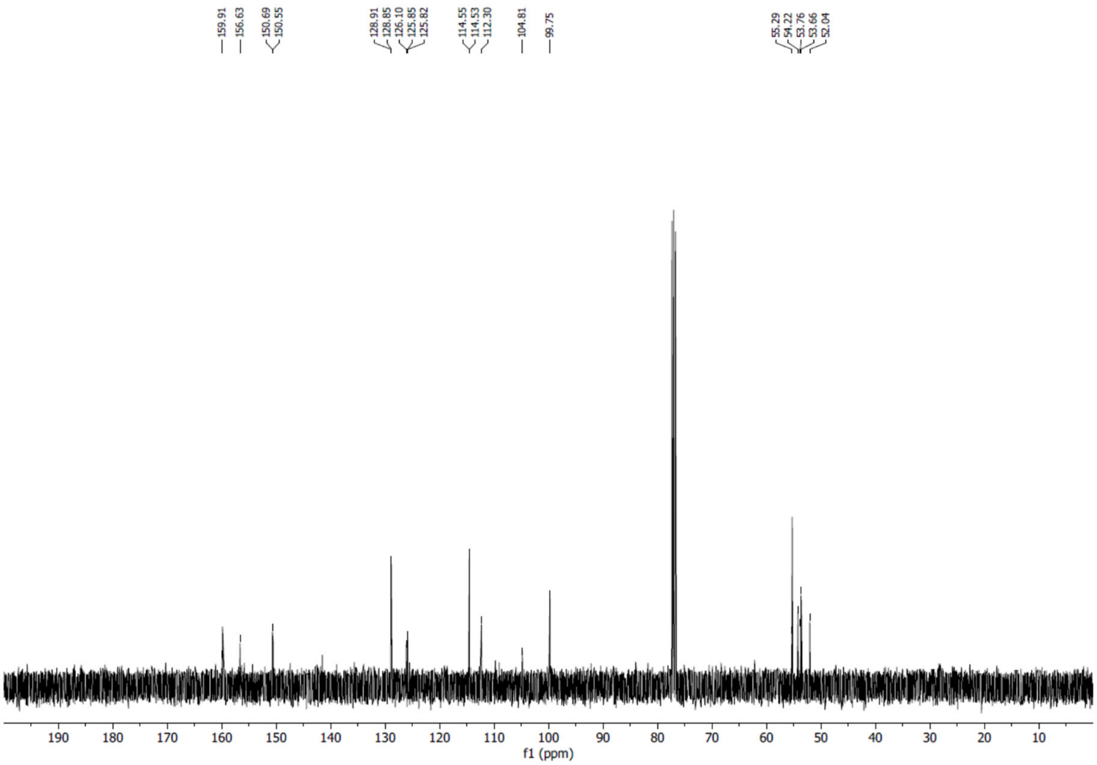

Figure S8. ^{13}C NMR (100 MHz, $CDCl_3$) of (**2**).

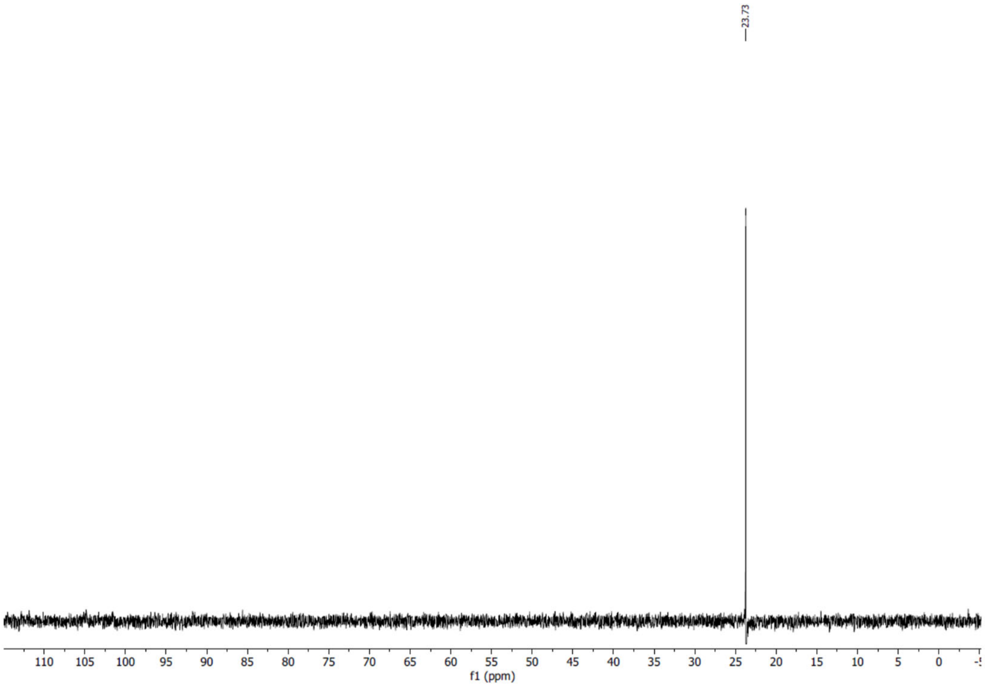

Figure S9. ^{31}P NMR (162 MHz, $CDCl_3$) of (**2**).

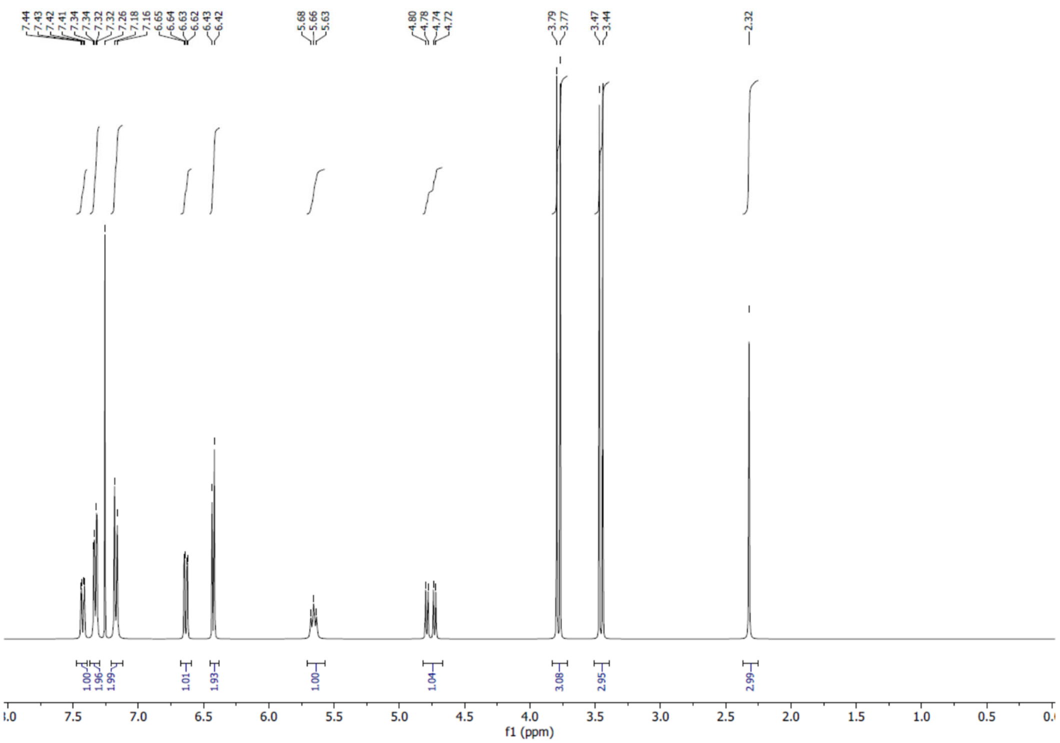

Figure S10. ^{1}H NMR (400 MHz, $CDCl_3$) of (**3**).

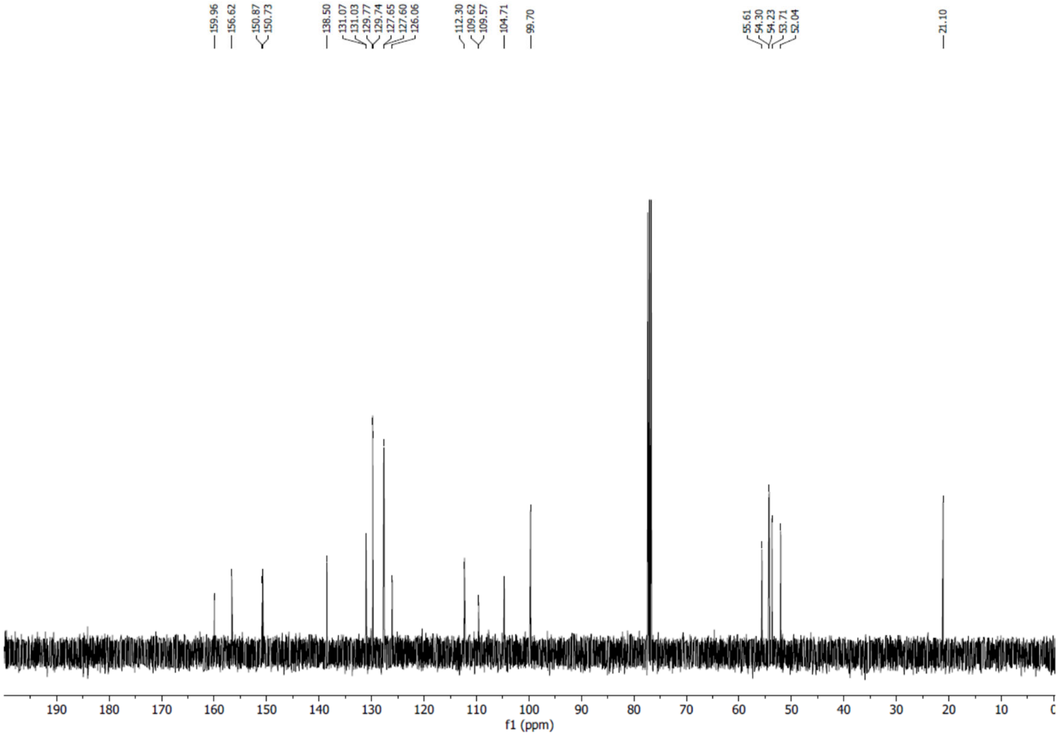

Figure S11. ^{13}C NMR (100 MHz, $CDCl_3$) of (**3**).

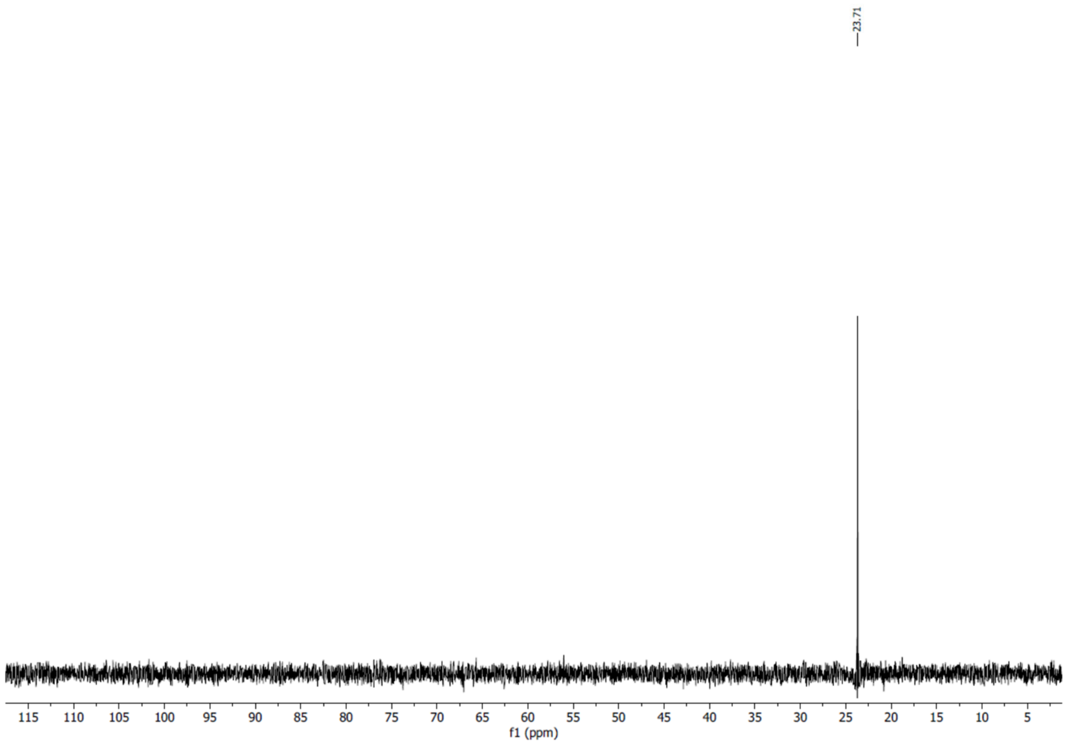

Figure S12. ^{31}P NMR (162 MHz, $CDCl_3$) of (**3**).

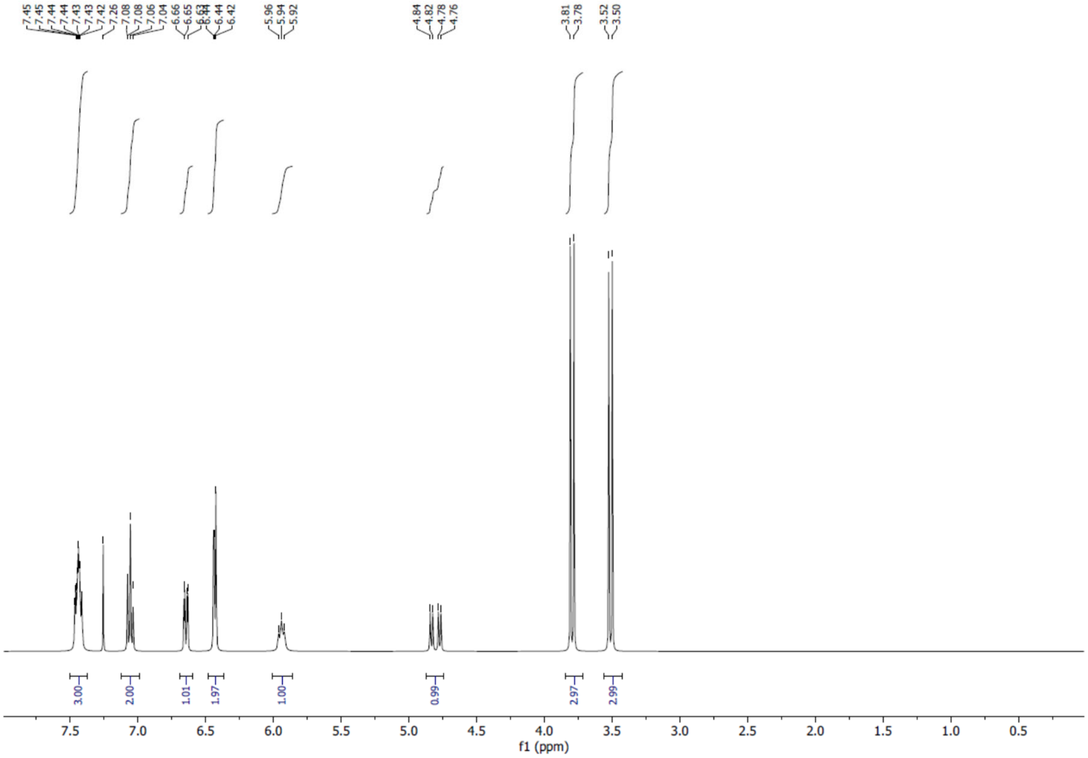

Figure S13. ^{1}H NMR (400 MHz, $CDCl_3$) of (**4**).

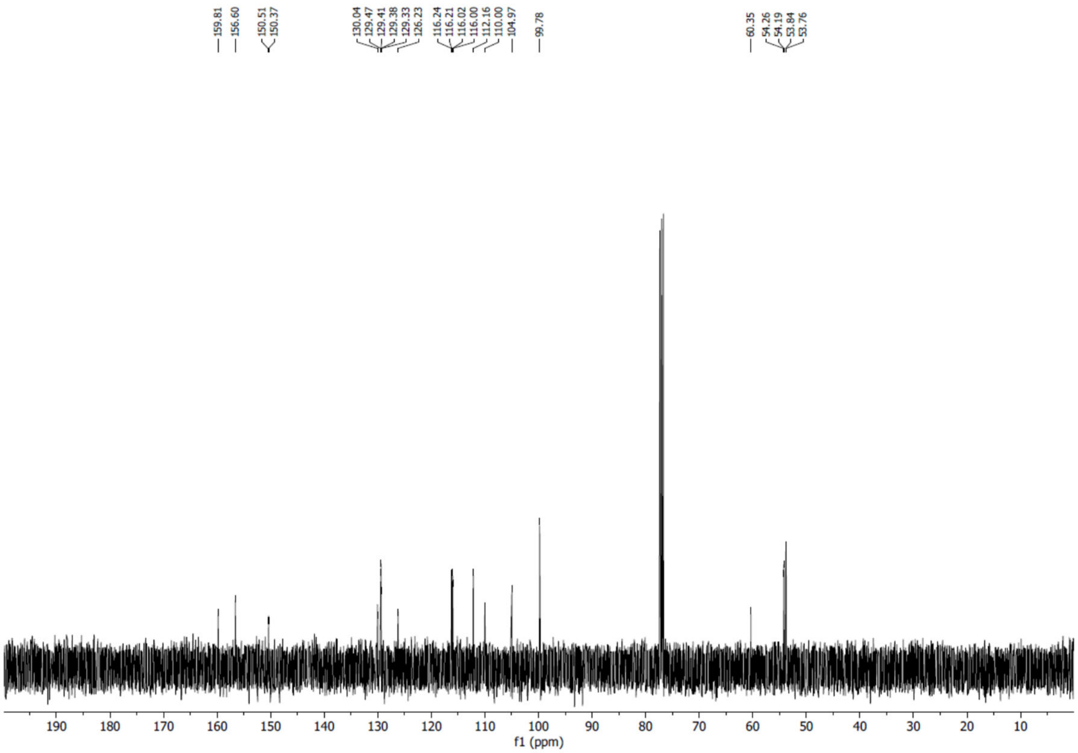

Figure S14. ^{13}C NMR (100 MHz, $CDCl_3$) of (**4**).

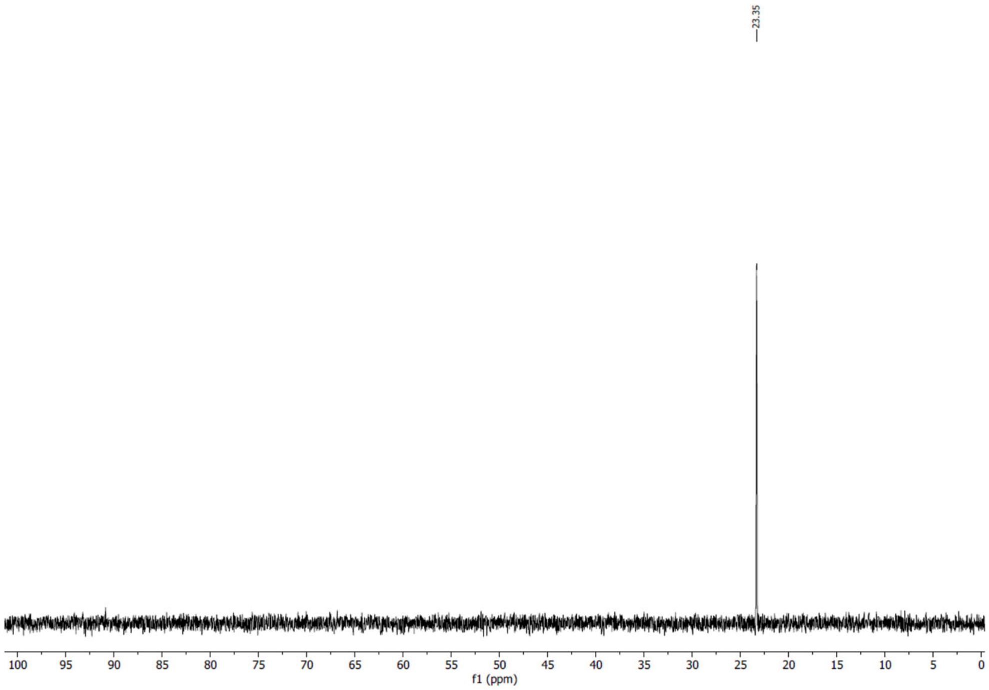

Figure S15. ^{31}P NMR (162 MHz, $CDCl_3$) of (**4**).

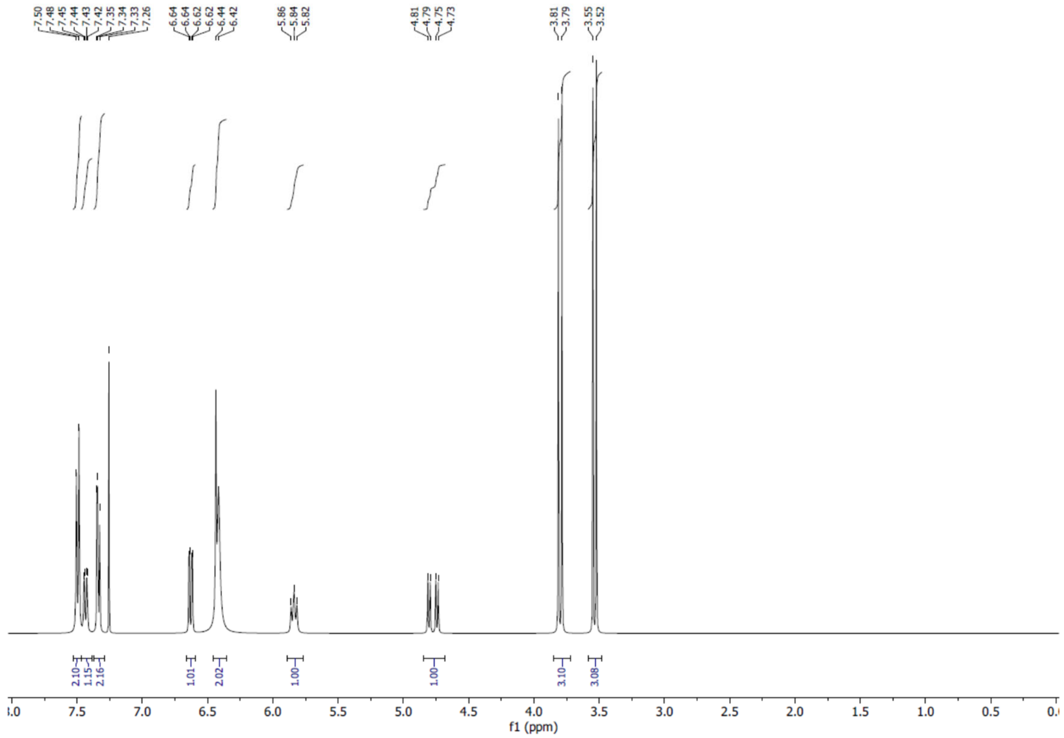

Figure S16. ^{1}H NMR (400 MHz, $CDCl_3$) of (**5**).

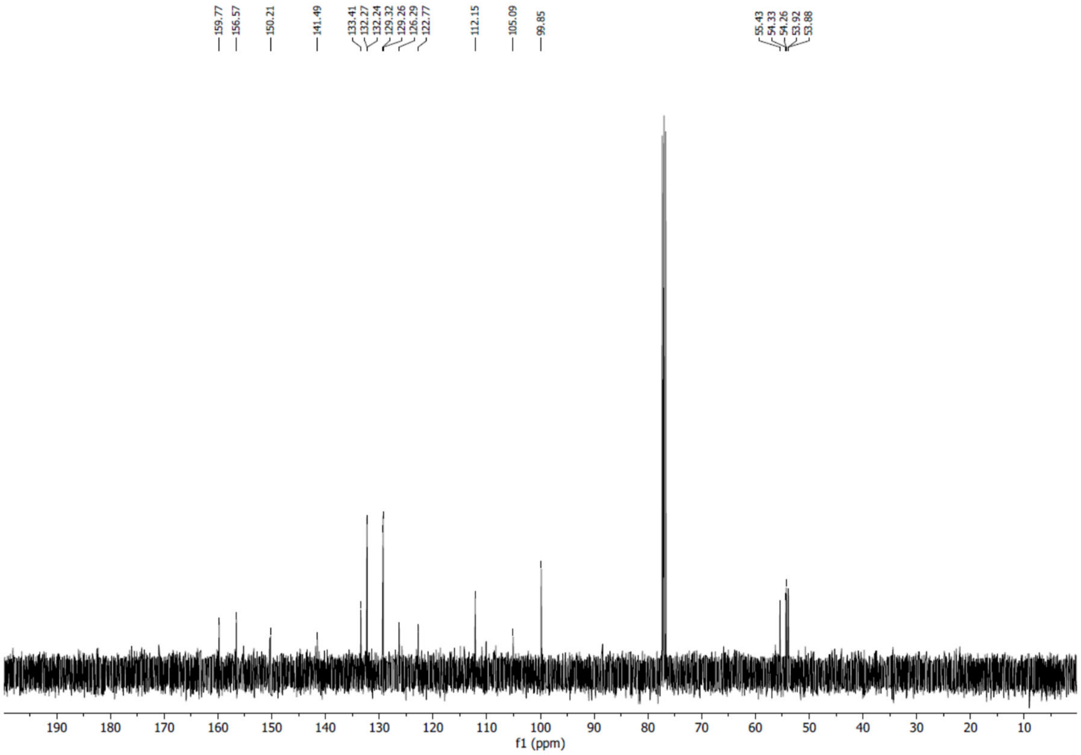

Figure S17. ^{13}C NMR (100 MHz, $CDCl_3$) of (**5**).

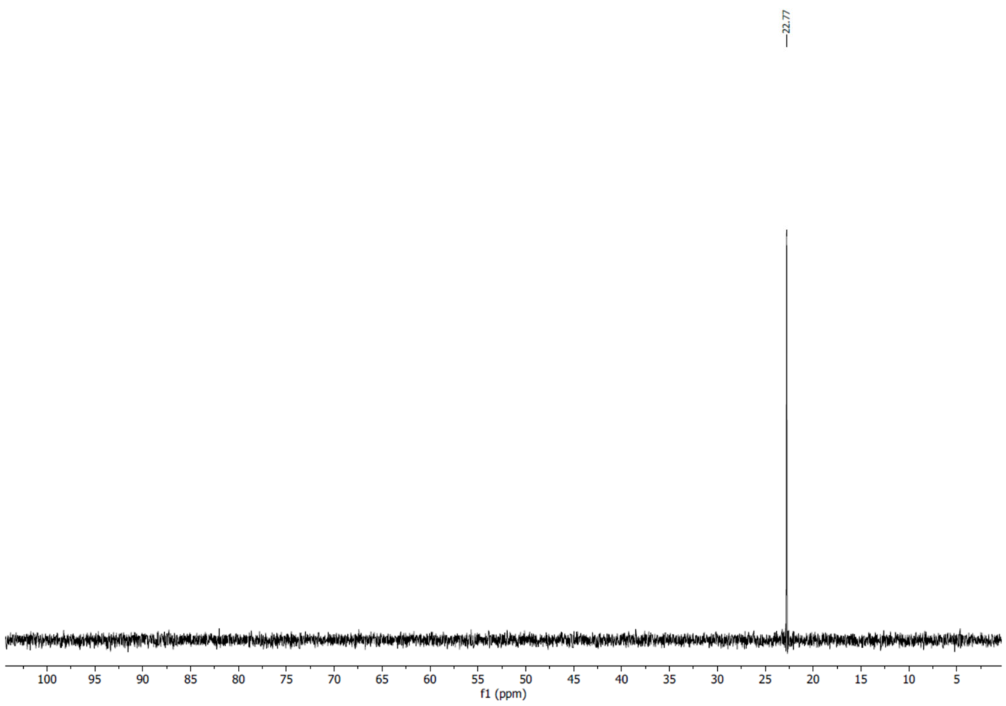

Figure S18. ^{31}P NMR (162 MHz, $CDCl_3$) of (**5**).

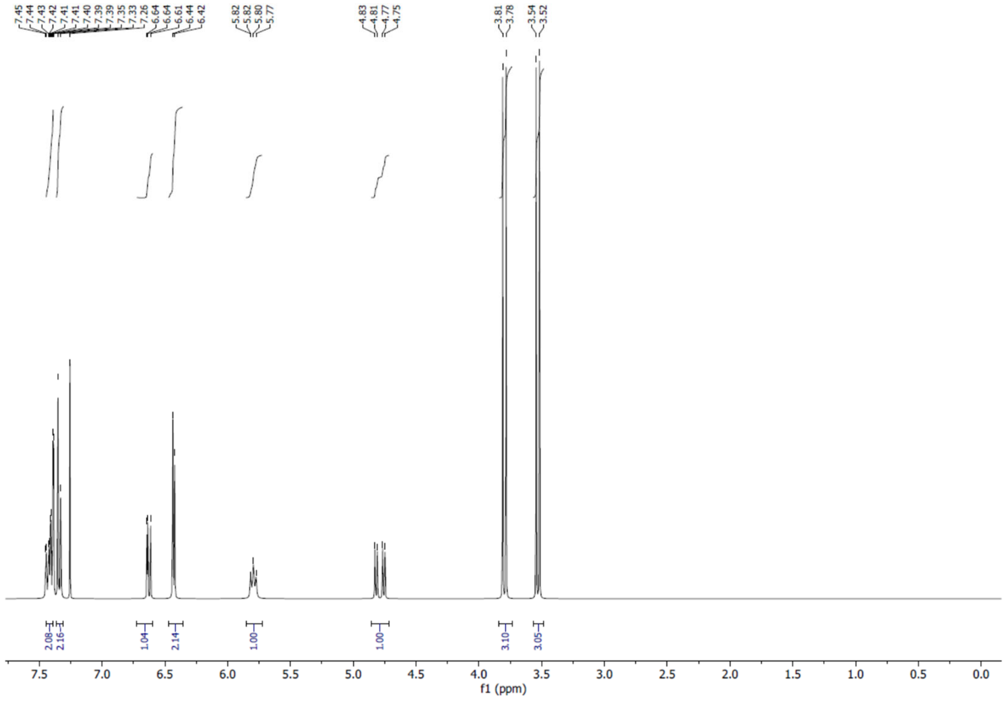

Figure S19. ^{1}H NMR (400 MHz, $CDCl_3$) of (**6**).

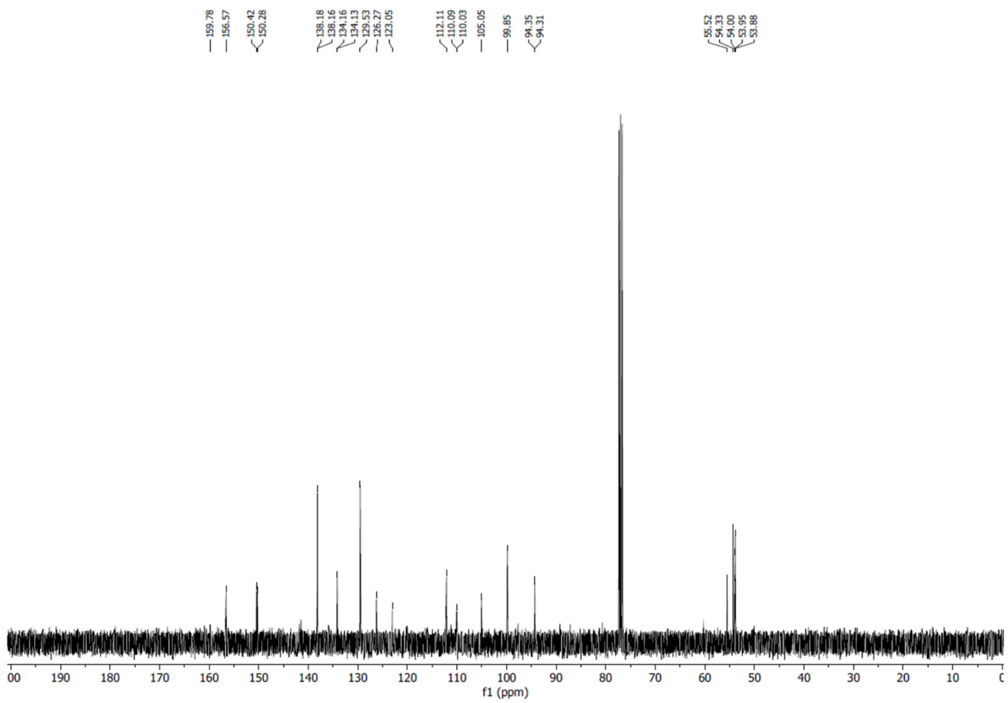

Figure S20. ^{13}C NMR (100 MHz, $CDCl_3$) of (**6**).

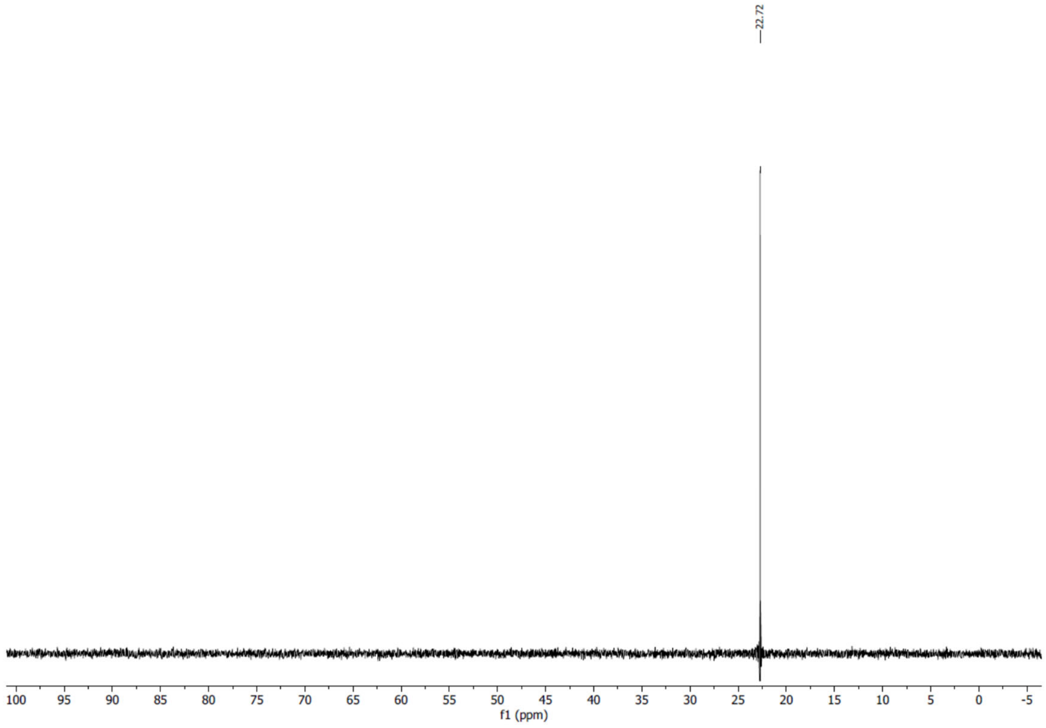

Figure S21. ^{31}P NMR (162 MHz, $CDCl_3$) of (**6**).

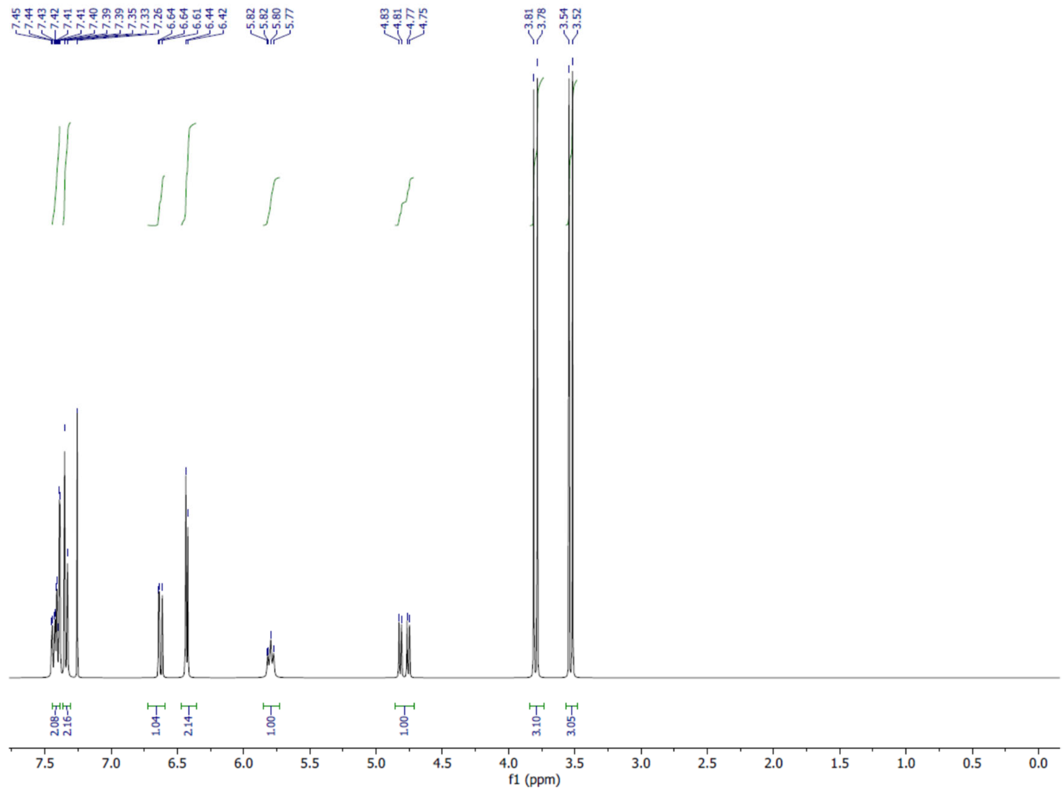

Figure S22. ^{1}H NMR (400 MHz, $CDCl_3$) of (**7**).

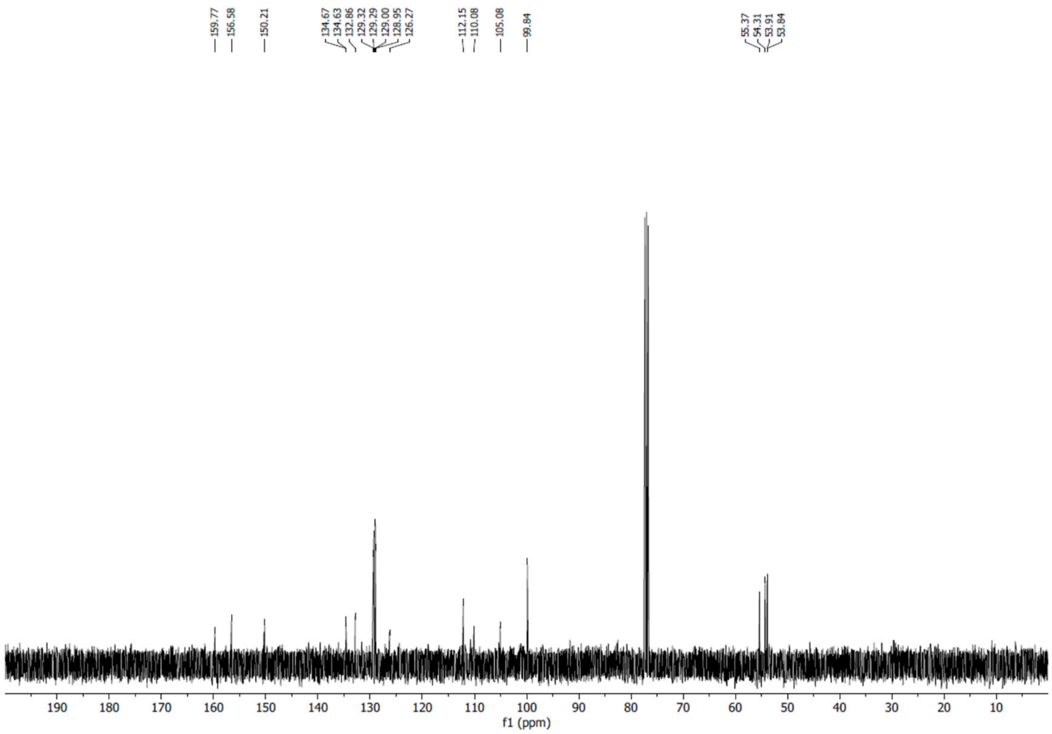

Figure S23. ^{13}C NMR (100 MHz, $CDCl_3$) of (**7**).

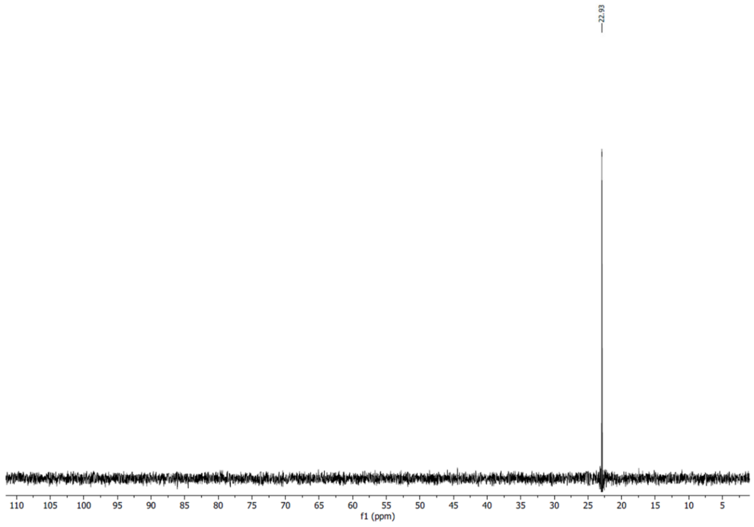

Figure S24. ^{31}P NMR (162 MHz, $CDCl_3$) of (**7**).

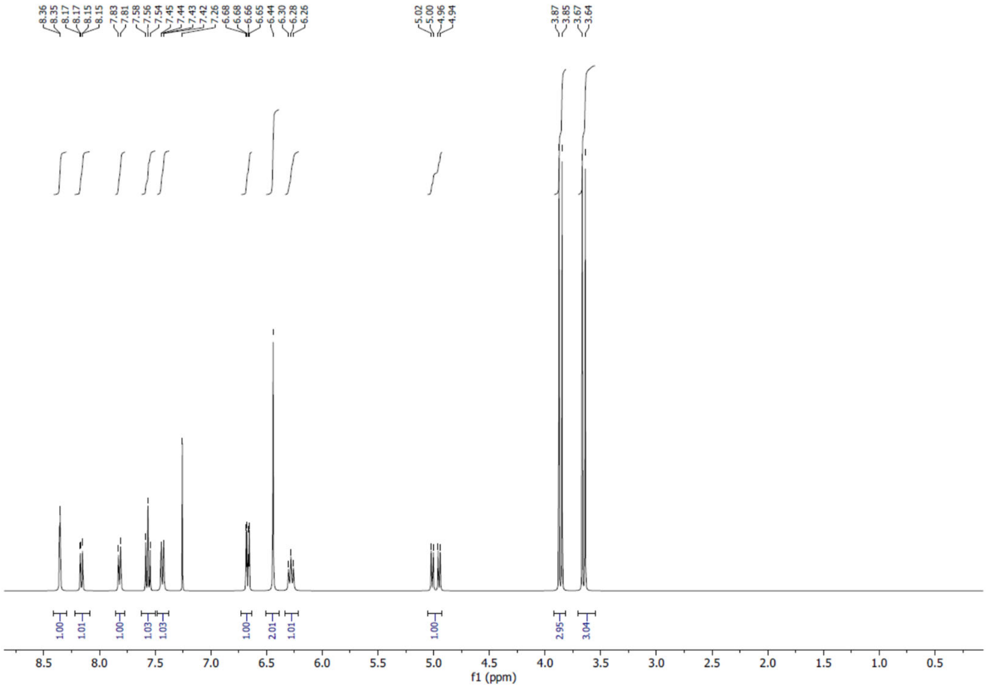

Figure S25. ^{1}H NMR (400 MHz, $CDCl_3$) of (**8**).

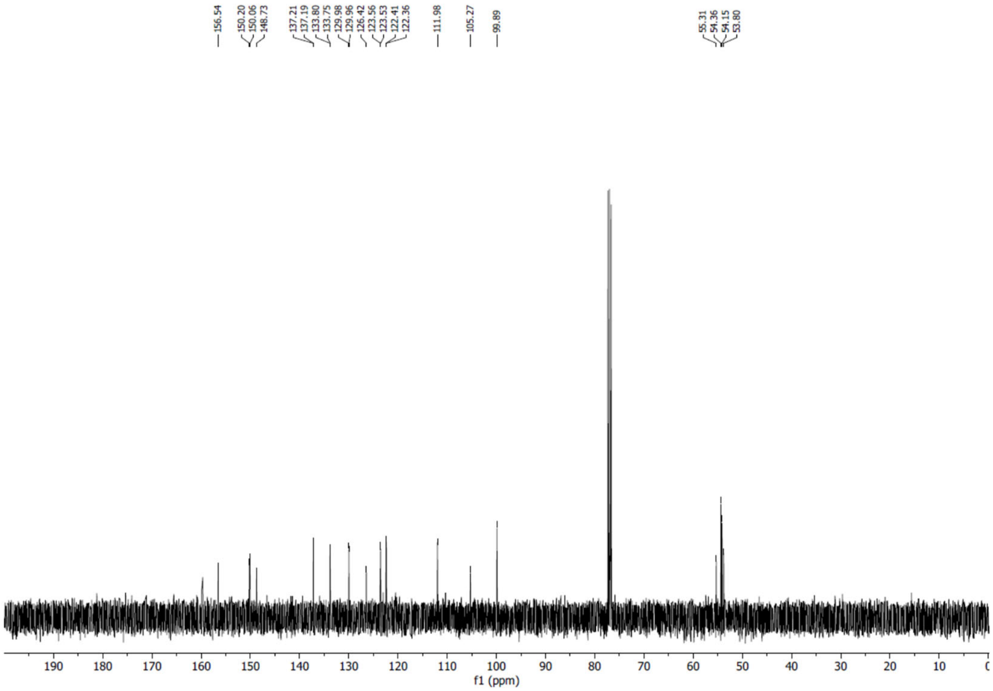

Figure S26. ^{13}C NMR (100 MHz, $CDCl_3$) of (**8**).

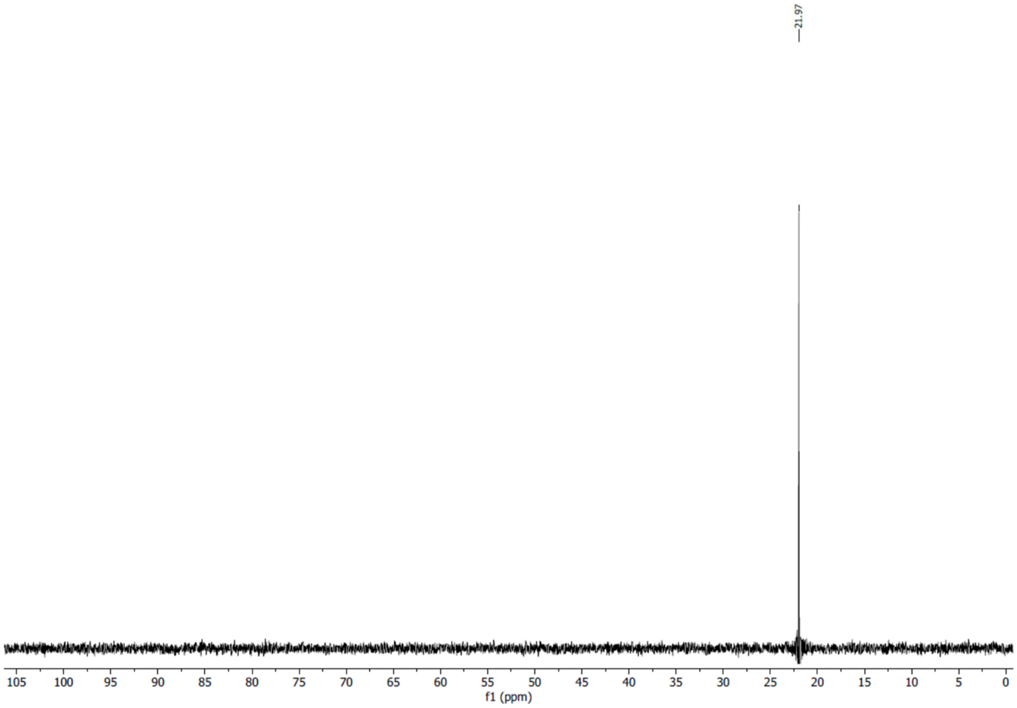

Figure S27. ^{31}P NMR (162 MHz, $CDCl_3$) of (**8**).

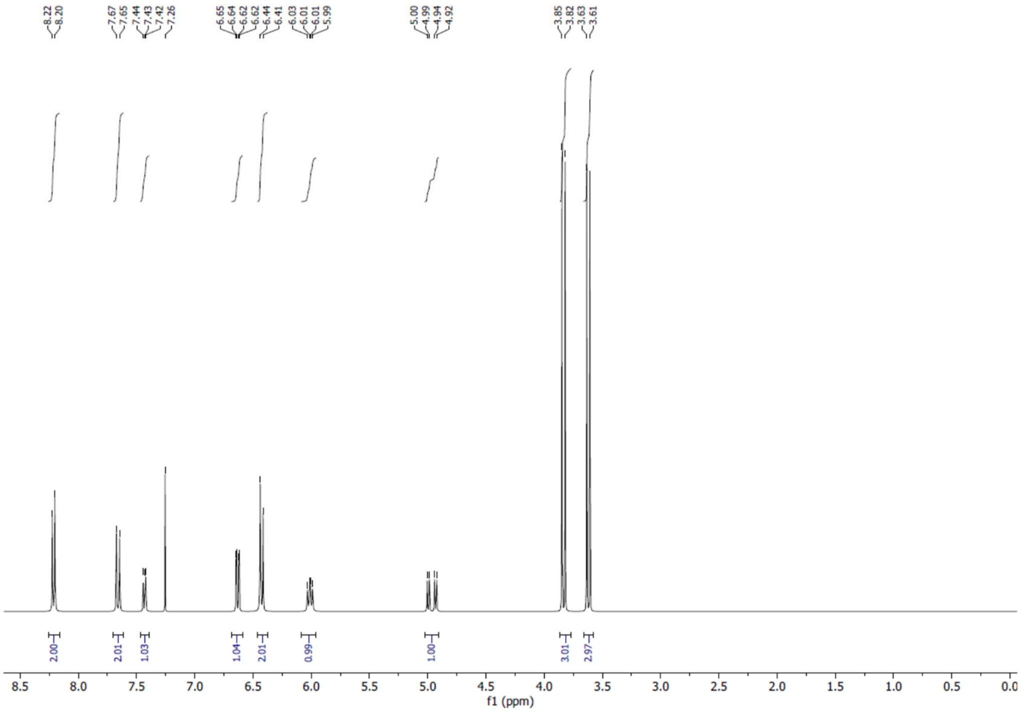

Figure S28. ^{1}H NMR (400 MHz, $CDCl_3$) of (**9**).

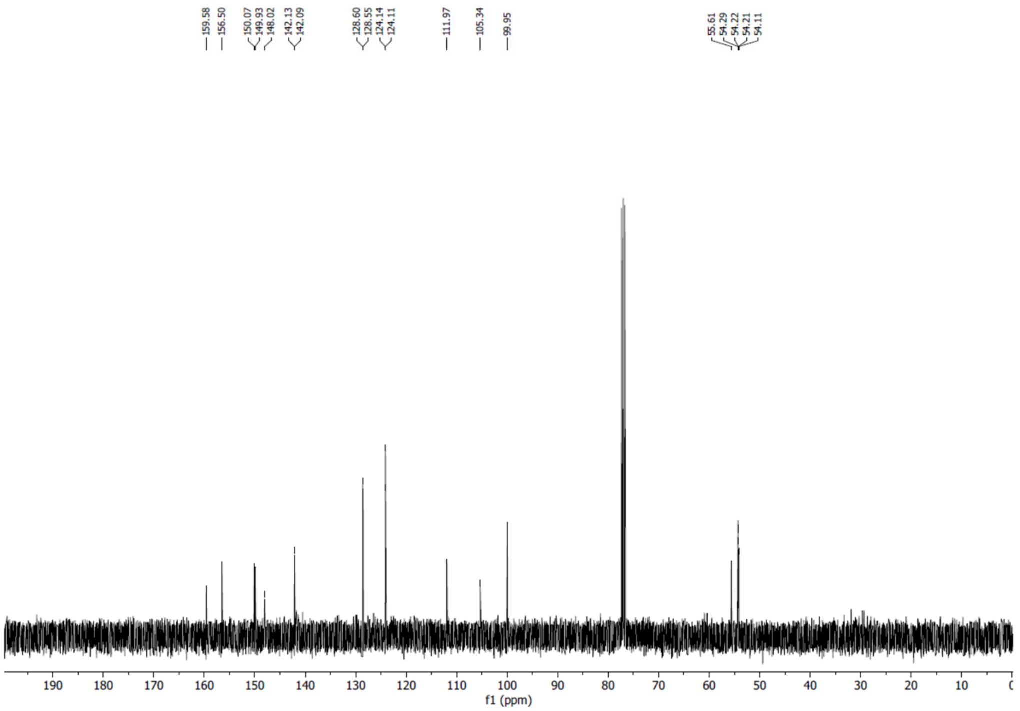

Figure S29. ^{13}C NMR (100 MHz, $CDCl_3$) of (**9**).

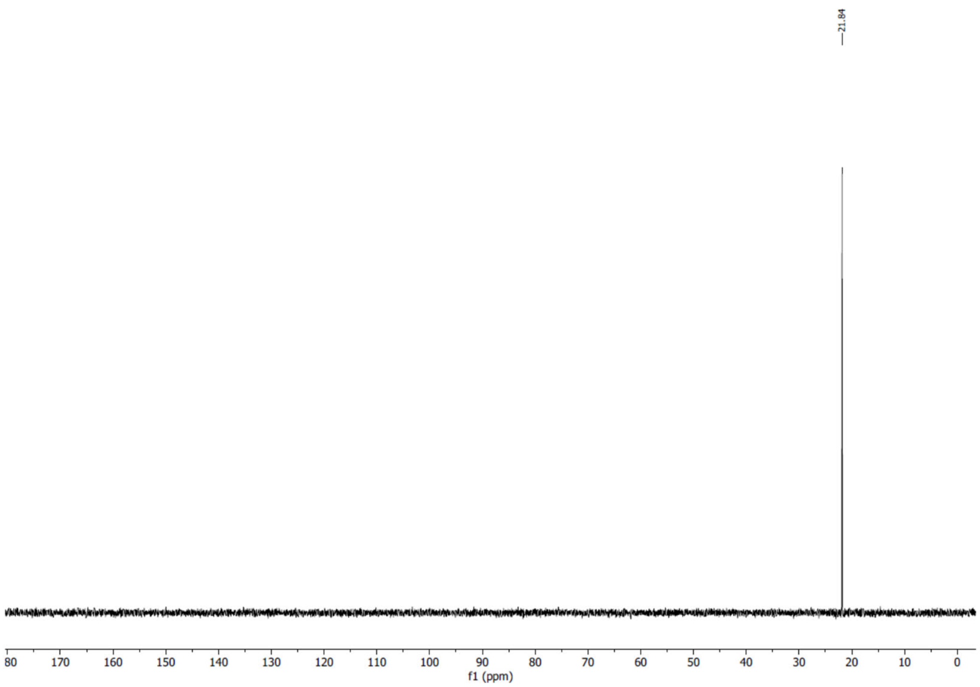

Figure S30. ^{31}P NMR (162 MHz, $CDCl_3$) of (**9**).

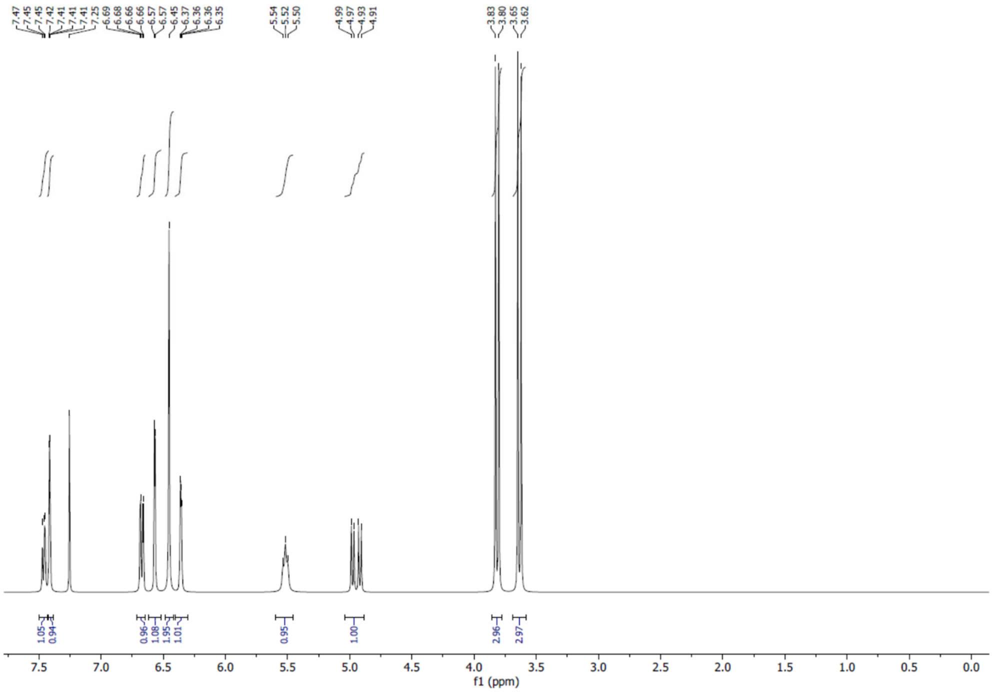

Figure S31. ^{1}H NMR (400 MHz, $CDCl_3$) of (**10**).

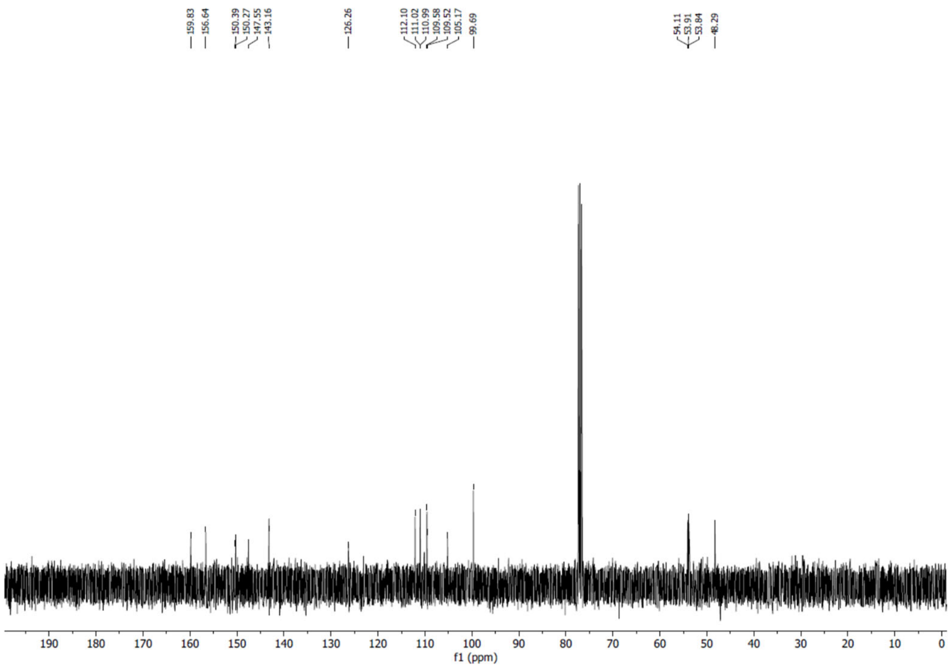

Figure S32. ^{13}C NMR (100 MHz, $CDCl_3$) of (**10**).

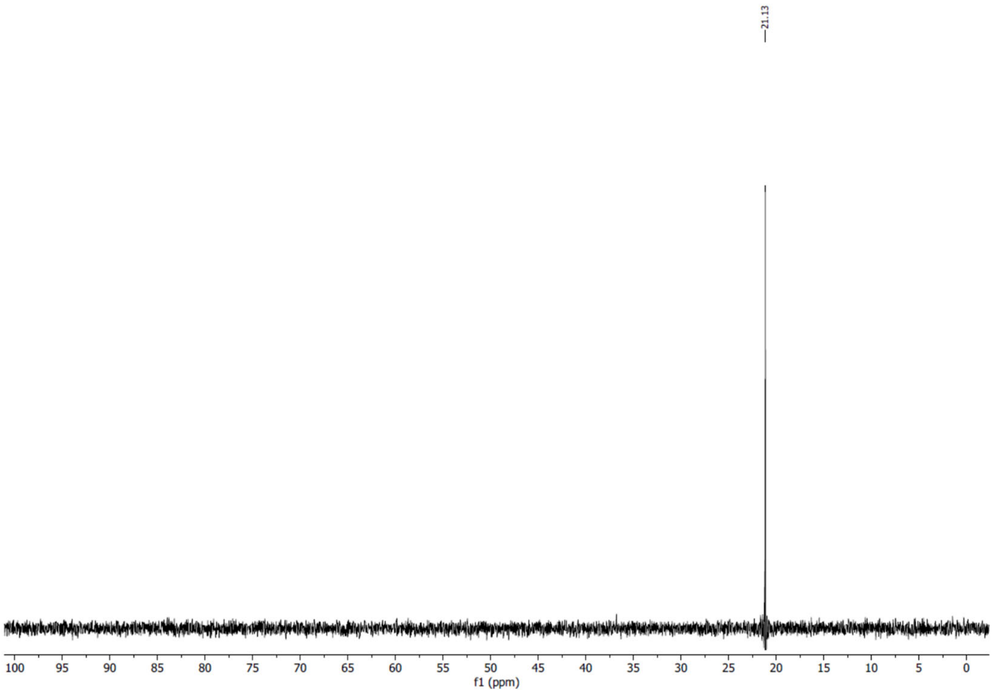

Figure S33. ^{31}P NMR (162 MHz, $CDCl_3$) of (**10**).

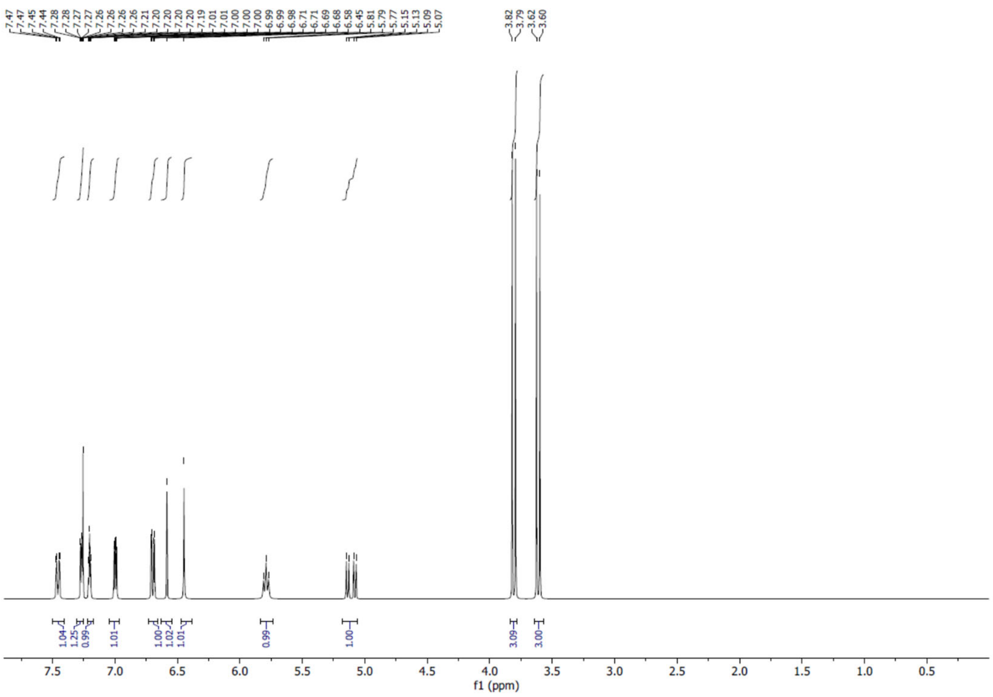

Figure S34. ^{1}H NMR (400 MHz, $CDCl_3$) of (**11**).

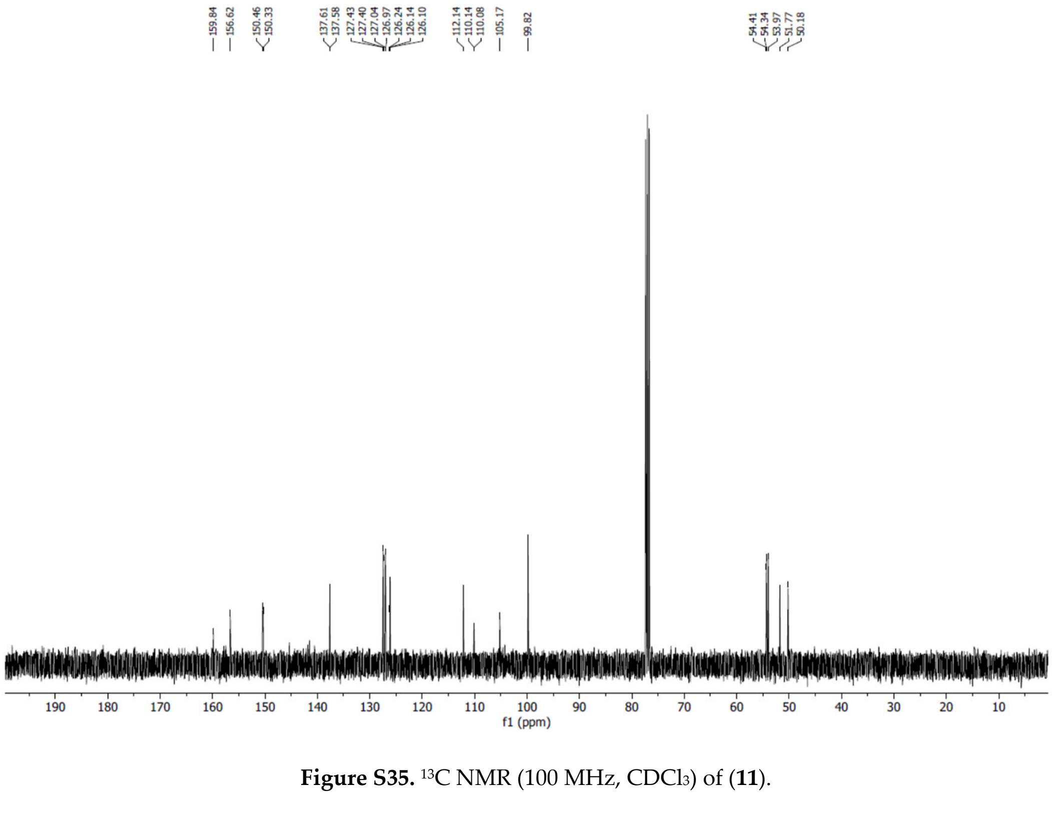

Figure S35. ^{13}C NMR (100 MHz, $CDCl_3$) of (**11**).

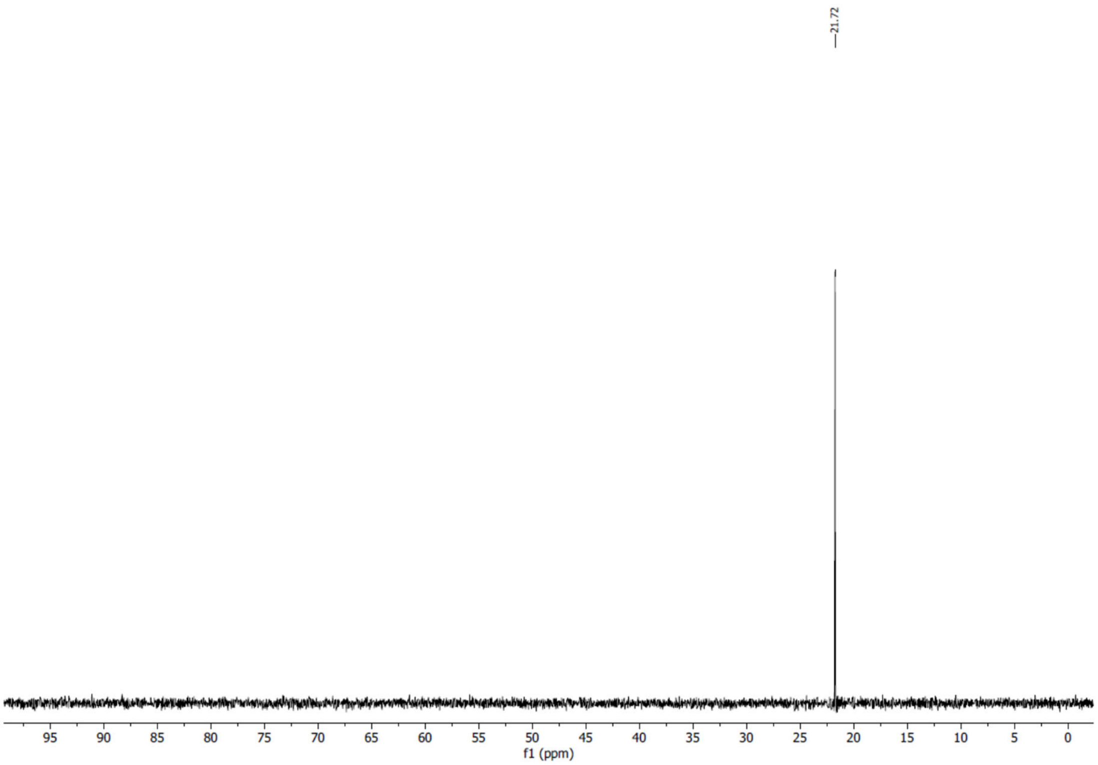

Figure S36. ^{31}P NMR (162 MHz, $CDCl_3$) of (**11**).

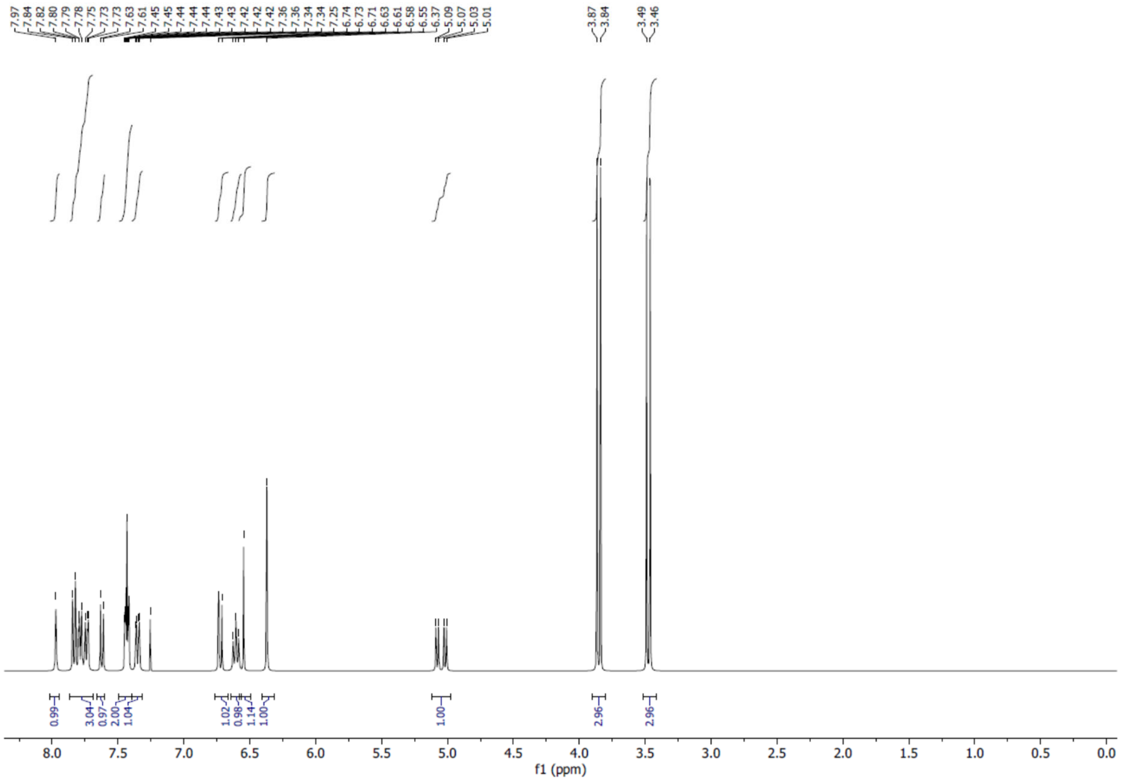

Figure S37. ^{1}H NMR (400 MHz, $CDCl_3$) of (**12**).

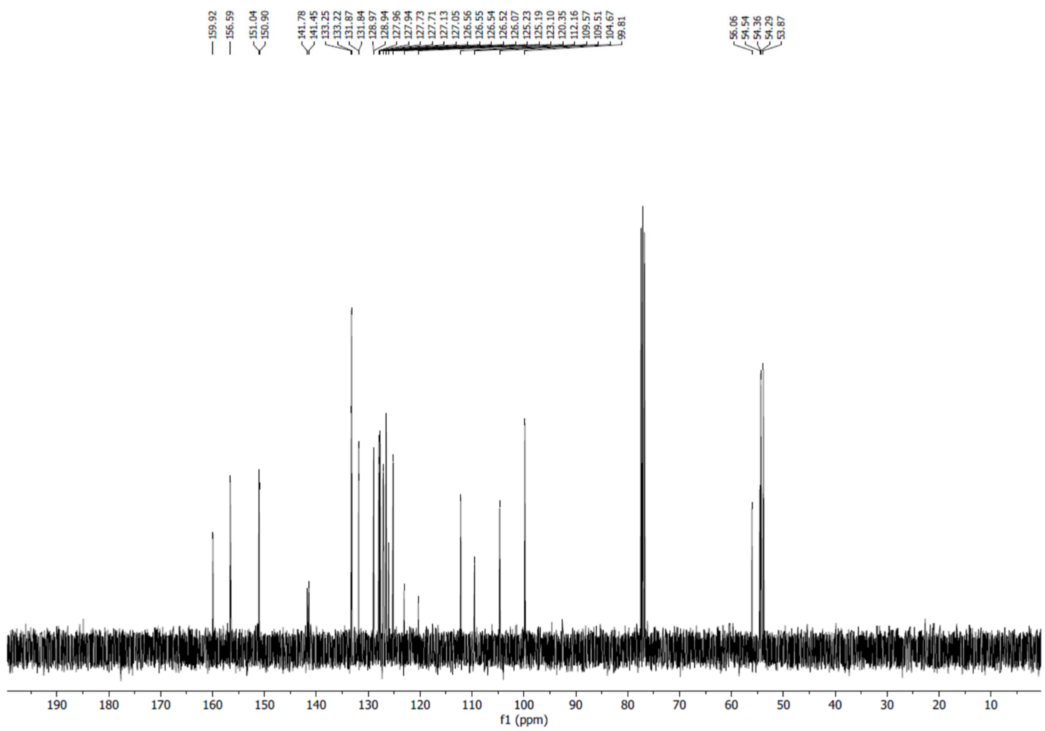

Figure S38. ^{13}C NMR (100 MHz, $CDCl_3$) of (**12**).

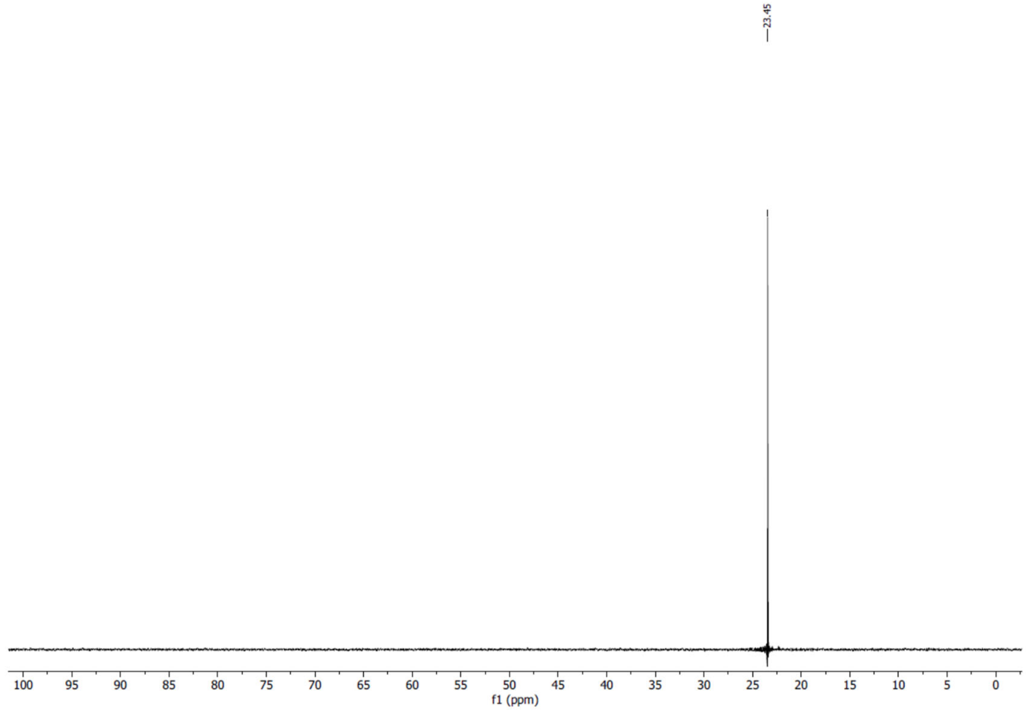

Figure S39. ^{31}P NMR (162 MHz, $CDCl_3$) of (**12**).

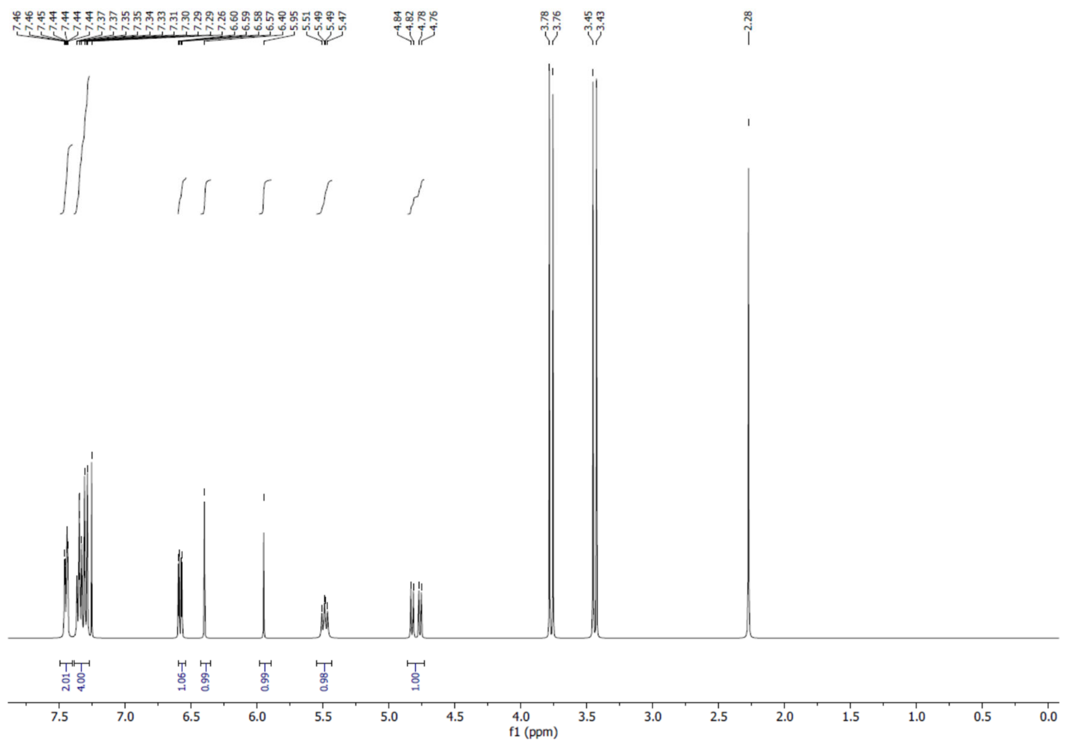

Figure S40. ^{1}H NMR (400 MHz, $CDCl_3$) of (**13**).

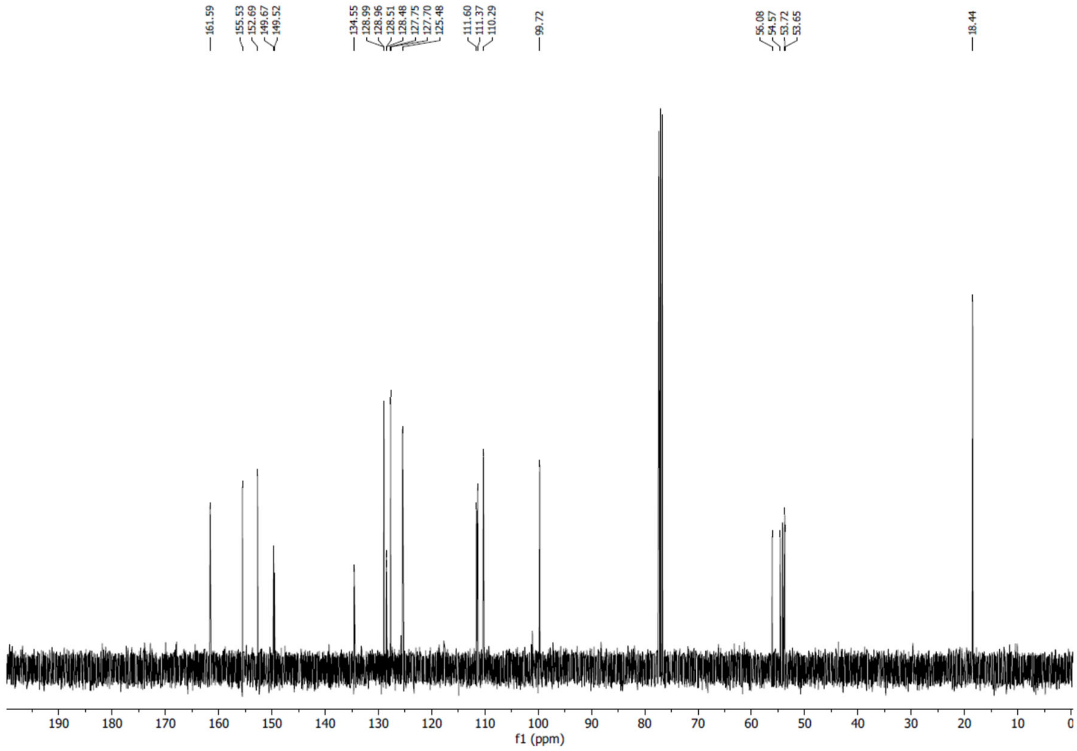

Figure S41. ^{13}C NMR (100 MHz, $CDCl_3$) of (**13**).

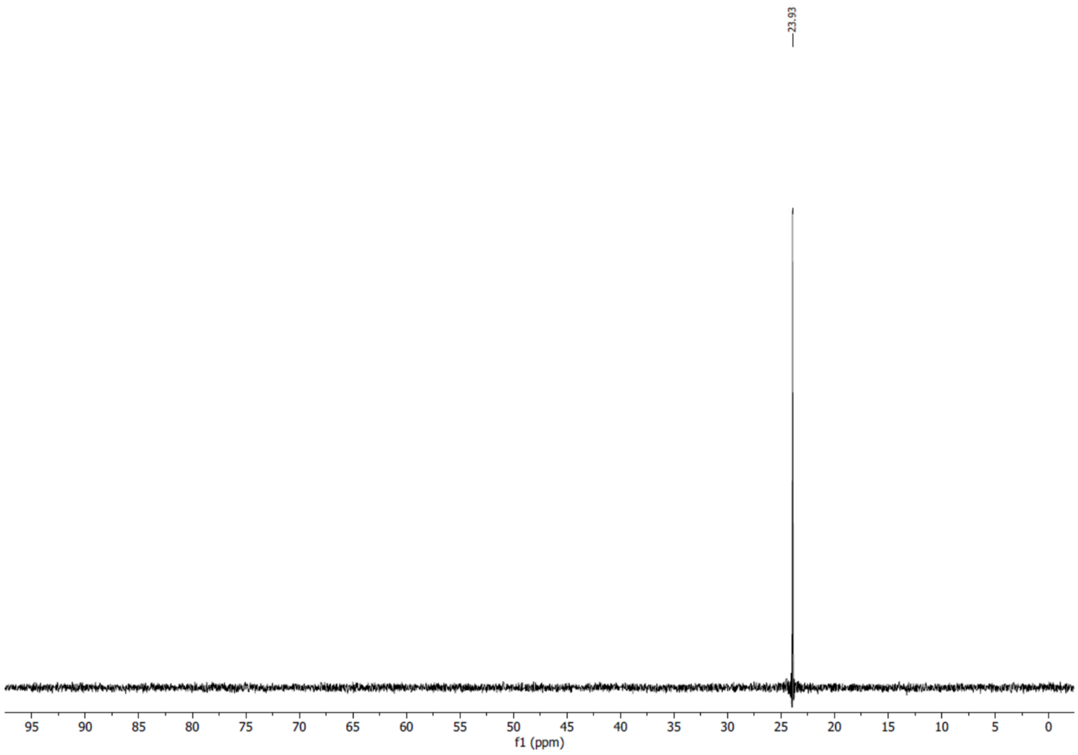

Figure S42. ^{31}P NMR (162 MHz, $CDCl_3$) of (**13**).

1.
